# Pomalidomide enhances the maturation of dendritic cells derived from healthy donors and multiple myeloma patients

**DOI:** 10.3389/fphar.2022.1076096

**Published:** 2022-12-05

**Authors:** Xi Wang, Jingying Dai, Jingyi Xia, Zichen Ye, Xiaobing Huang, Wanjun Cao, Rong Xiao, Lin He

**Affiliations:** ^1^ Sichuan Academy of Medical Sciences and Sichuan Provincial People’s Hospital, School of Medicine, University of Electronic Science and Technology of China, Chengdu, China; ^2^ Department of Pharmacy, Nanchong Central Hospital, Nanchong, China

**Keywords:** pomalidomide, multiple myeloma, monocyte-derived dendritic cells, maturation, DC adjuvant

## Abstract

**Objective:** To explore the effect of pomalidomide on the maturation of monocyte-derived dendritic cells (moDCs) from healthy donors (HDs) and multiple myeloma (MM) patients.

**Methods:** MoDCs were generated by the incubation of monocytes from peripheral blood mononuclear cells (PBMCs) for 7 days in a medium consisting of 800 U/ml granulocyte-macrophage colony stimulating factor (GM-CSF), 500 U/ml interleukin-4 (IL-4), RPMI 1,640 medium, 5% human serum, 100 U/ml penicillin and 0.1 mg/ml streptomycin. Meanwhile, the incubation system was administrated with 10 µM pomalidomide or 1 × PBS as the control group. On the eighth day, cells were harvested and analyzed by flow cytometry. The CD80^+^CD86^+^ cell population in total cells was gated as moDCs in the FACS analyzing system. After that, the expression of CD40 and HLA-DR on moDCs was analyzed. Meanwhile, the supernatant from the incubation system was evaluated for the secretion of cytokines interleukin-12 (IL-12), tumor necrosis factor-α (TNF-α), and macrophage inflammatory protein 1α (MIP-1α) by enzyme-linked immunosorbent assay (ELISA).

**Results:** When analyzing all the HD-moDCs together (*n* = 15), pomalidomide significantly increased the mean fluorescence intensity (MFI) of CD40 expression and HLA-DR expression on moDCs compared with the control group (*p* = 0.003, *p* = 0.040). Meanwhile, the proportion of CD40^+^ moDCs and HLA-DR^+^ moDCs in total moDCs was significantly higher in the pomalidomide group than in the control group (*p* = 0.008, *p* = 0.032). When analyzing all MM patient-moDCs together (*n* = 11), pomalidomide significantly increased the MFI of CD40 expression and HLA-DR expression on moDCs compared with the control group (*p* = 0.047, *p* = 0.006). Meanwhile, the proportion of HLA-DR^+^ moDCs in total DCs was significantly higher in the pomalidomide group than in the control group (*p* < 0.001). Moreover, HD-moDCs (*n* = 8) treated with pomalidomide secreted 192% IL-12, 110% TNF-α, and 112% MIP-1α of the untreated moDCs (*p* = 0.020, *p* = 0.006, *p* = 0.055). However, when analyzing MM patient-moDCs (*n* = 10) together, the secretion of IL-12, TNF-α and MIP-1α from moDCs showed no significant difference between the pomalidomide group and the control group (*p* = 0.458, *p* = 0.377, *p* = 0.248).

**Conclusion:** *In vitro*, 10 µM pomalidomide enhances the maturation of moDCs derived from both HDs and MM patients. Pomalidomide shows potential to be applied as a DC adjuvant for DC-based immunotherapy, such as the DC vaccine and DC cell therapy in MM.

## Introduction

Multiple myeloma is a malignant disease with plasma cell origin, and the incidence of which ranks second in the hematological malignancies (Pinto et al., 2020). MM is still an incurable disease with high recurrence. The pathogenesis of MM is closely relevant to the chromosome deletion, the gene abnormity, and the change of immune microenvironment. Furthermore, immune disorders occur in the early stage of MM (Walker et al., 2018). The immunotherapy strategy which aims to reverse the immune disorders and improve the patients’ anti-myeloma immunity can effectively eliminate cancer cells *in vivo* by improving immune surveillance, activation and killing. Thus, immunotherapy is able to achieve a favorable clinical efficacy. Dendritic cells (DCs) play an essential role in initiating the anti-cancer immune response. As the most critical antigen-presenting cell (APC), DCs present major histocompatibility complex (MHC) molecules and tumor antigens to prime naïve T cells. Meanwhile, DCs provide costimulatory signals to T cells for the further activation of T cells (Wang et al., 2016). Thus, DCs act as the most critical first-step in initiating the anti-cancer specific immune response.

It has been found that MM patients show significant immunodeficiency. The immunodeficient DCs of MM patients is one of the main reasons why specific anti-tumor immunity can’t be normally and effectively activated (Li and Wang, 2019; Tamura et al., 2019), which leads to the occurrence and development of MM. Therefore, reversing the severe immunodeficiency of DCs in MM patients to improve the activity and maturity of DCs is one of the critical problems to be solved. In recent years, the generation of autologous DCs of MM patients *in vitro* with enhanced activity and maturity has been applied for DC cell therapy in MM patients. This immunotherapy strategy is the frontier research field worldwide with a promising translational future. Although DC immunotherapy has been developing in the past decades, the techniques of *in vitro* generation of DC, which to a certain extent enhance the activity and maturity of patient DC still show a distance from achieving significant clinical efficacy with successful generation of ideally active and mature DC of patients. Therefore, improving the activity and maturity of DCs of MM patients generated *in vitro* to a normal or significantly enhanced level becomes the essential issue to be solved in DC immunotherapy for MM.

Pomalidomide is the third generation of immunomodulatory drugs (IMiDs) as an analogue of thalidomide and lenalidomide. Pomalidomide has not only been approved for treating relapsed/refractory MM due to its direct anti-tumor effect (Rajkumar and Kumar, 2016) but also shows a significant immunomodulatory effect on immune cells. On the one hand, pomalidomide exerts the direct anti-myeloma effect by up-regulating the expression of P21^waf1^ (Escoubet-Lozach et al., 2009), down-regulating the expression of C/EBP *β* (Li et al., 2011) or activating the caspase 8 (Mitsiades et al., 2002). On the other hand, it has been demonstrated that a combination of pomalidomide and low-dose dexamethasone can activate T cell function by increasing the release of interferon *γ* (IFN-γ), TNF-α, interleukin-2 (IL-2) derived from the T cells of relapsed/refractory MM patients. In addition, the release of TNF-α, IFN-γ, perforin and granzyme from the NK cells, the expression of CD16, adhesion molecule CD11a on NK cell surface, and the antibody-dependent cell-mediated cytotoxicity effect (ADCC) of NK cells were all enhanced with this regimen (Sehgal et al., 2015). It has been demonstrated that 10 μM pomalidomide inhibits regulatory T cells (Tregs) of HDs *in vitro* based on downregulation of the Foxp3 gene’s expression. Detailly, the proportion of CTLA-4^+^FOXP3^+^ cells in PBMCs is decreased (Galustian et al., 2009; Bila et al., 2021). Moreover, Henry et al. (2013) have demonstrated that 10 μM pomalidomide significantly increases the antigen uptake of DCs. However, the immunomodulatory effect of pomalidomide on human DCs has not been deeply investigated and clarified. Therefore, we investigated the immunomodulatory effect of pomalidomide on moDCs derived from HDs and MM patients, focusing on the maturation of moDCs in this study.

## Materials and methods

### Donors

The study has gained approval from the Ethics Committee of the Sichuan Provincial People’s Hospital. All the donors enrolled in this study have read and signed the informed consents. This study included 15 HD donors (10 females and 5 males) and 11 MM patient donors (4 females and 7 males). All the donors should be 18–70 years old. MM patient donors should achieve partial response (PR) or complete response (CR) after treatment. The patient donors should not have immune-related diseases. In addition, the patient donors should not be undergoing chemotherapy at the time of peripheral blood donation.

### Materials

Pomalidomide and human AB serum were purchased from Sigma, United States. RPMI 1,640 medium was purchased from Gibco, United States. GM-CSF and IL-4 were purchased from Novoprotein, China. Penicillin-streptomycin solution was purchased from Hyclone, United States. Human Lymphocyte separation medium (Ficoll) was purchased from GE Lifesciences, United States. 1×PBS and DMSO were purchased from Solarbio, China. FITC anti-human CD80 mAb, PE anti-human CD86 mAb, APC anti-human CD40 mAb and Pacific Blue anti-human HLA-DR mAb were all purchased from Biolegend, China.

### Acquisition of peripheral blood mononuclear cells

16 ml of peripheral blood was gained from HDs (*n* = 15) and MM patients (*n* = 11) after the donors read and signed the informed consents. PBMCs were isolated by density gradient centrifugation. Specifically, equal volume of 1 × PBS was used to dilute the peripheral blood. Then the diluted peripheral blood was slowly added to the top of Ficoll for 30 min of centrifugation (400 × *g*). PBMCs were collected and washed with 1 × PBS for 3 times.

### Dendritic cell generation

PBMCs were evenly divided into two groups and cultured at 5 × 10^6^ cells/ml in RPMI 1,640 medium at 37°C in air containing 5% CO2. After 3 h of incubation, monocytes were isolated by adhesion, which were cultured in a medium consisting of 800 U/ml GM-CSF, 500 U/ml IL-4, RPMI 1,640 medium, 5% human serum, 100 U/ml penicillin and 0.1 mg/ml streptomycin for 7 days at 37°C in air containing 5% CO_2._ 10 µM pomalidomide was given to one group while 1 × PBS was given to the other group as the control group. On the eighth day, moDCs were harvested. Moreover, the morphology of moDCs was observed by the inverted microscope.

### Immunophenotyping by flow cytometry

The expression of DC maturation-related surface makers CD40, HLA-DR, CD80, and CD86 were analyzed by flow cytometry. Pomalidomide or 1 × PBS treated moDCs were resuspended in 100 µl cold 1 × PBS, which was then co-incubated with APC-labeled CD40 mAb, Pacific Blue-labeled HLA-DR mAb, FITC-labeled CD80 mAb and PE-labeled CD86 mAb for 30 min at 4°C. Then cells were washed with cold 1 × PBS twice. At last, the cells were resuspended in 200 µl 1 × PBS for detection by flow cytometer.

**FIGURE 1:**
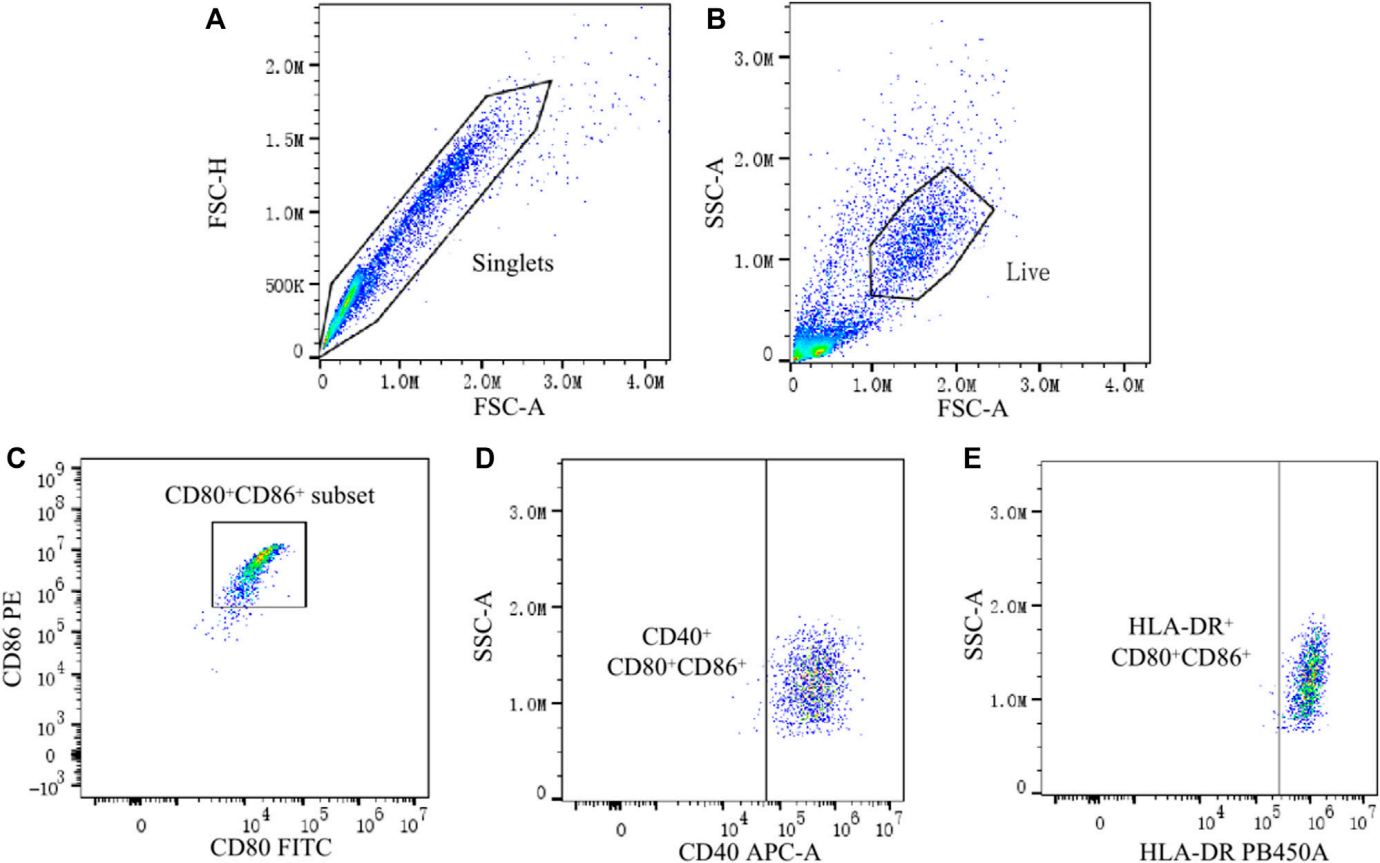
Analysis of CD40 and HLA-DR expression on moDCs by flow cytometry. **(A)** Single cells were gated by the Forward Scatter area (FSC-A) and Forward Scatter height (FSC-H). **(B)** Live cells from the single cells were further gated by the FSC-A and Side Scatter area (SSC-A). **(C)** CD80^+^ CD86^+^ cells (moDCs) were gated from live cells. **(D,E)** Finally, the expression of CD40 and HLA-DR on CD80^+^ CD86^+^ cells were analyzed.

The technique for generating moDCs in this study is a usually used and efficient technique, which can successfully generate moDCs from PBMCs (Büll et al., 2017; Sung, 2019). After the process for generating moDCs, there should be a big amount of moDCs in the harvested cells. CD80 and CD86 are typical surface markers of DCs, which are usually used for defining DCs (Xue et al., 2016; Liu et al., 2018). And we found that the CD80^+^ CD86^+^ cell population accounted for 74.0%–99.6% of the total cells (removal of adhered cells, cell debris and dead cells). Therefore, the CD80^+^ CD86^+^ cell population was regarded as moDCs in this study. Then, the expression of CD40 and HLA-DR on moDCs was analyzed. To be specific, the proportion of CD40^+^ moDCs and HLA-DR^+^ moDCs in total moDCs as well as the MFI of CD40 and HLA-DR on moDCs were analyzed respectively. Data was analyzed by FlowJo 10.4 software (Figure 1).

### Cytokine assays by ELISA

The secretion of DC maturation/activity-related cytokines IL-12, TNF-α and MIP-1α was analyzed by ELISA. Supernatant from the incubation system of pomalidomide or 1 × PBS treated moDCs was collected by centrifugation. The concentration of the cytokines IL-12, TNF-α, and MIP-1α in the supernatant was detected by the SEA ELISA kit following the manufacturer’s directions. Supernatant from the incubation system of 8 HD-moDCs and 11 MM patient-moDCs were evaluated. The other supernatant samples were not able to be evaluated as a result of the amount, contamination and experimental mistakes.

### Statistical analysis

Statistical analysis was performed with SPSS 17.0 software. Graphs were generated by Graphpad Prism 8.3.0 software. The normality of data distribution was determined with the Kolmogorov-Smirnov normality test. Paired *t*-test was used to compare whether there were differences in the expression of cytokines secreted by moDCs and the expression of DC maturation-related surface makers on moDCs administrated with or without pomalidomide. Independent *t*-test was used to compare whether there were differences in moDCs between the MM patient group and the HD group. Differences were considered to be significant if the *p*-value was<0.05 (*p < 0.05, **p < 0.01, ***p < 0.001). All data was expressed as Mean ± Standard Deviation (SD).

## Results

### The morphology of moDCs

**FIGURE 2:**
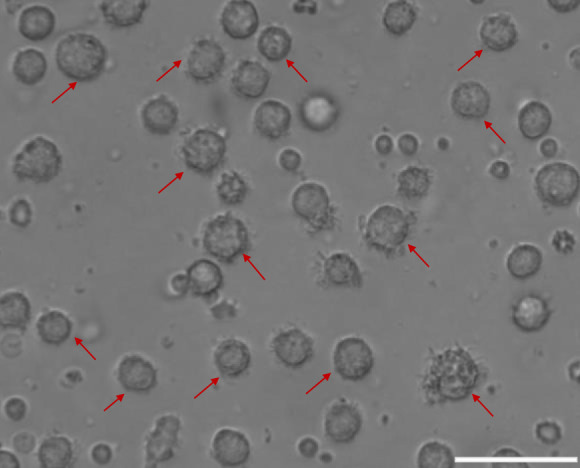
The morphology of moDCs. MoDCs are shown by the red arrow (100 μm).

On the eighth day, the morphology of moDCs was observed by the inverted microscope. As shown in Figure 2, the morphology of moDCs is large and irregular. There are apparent protrusions on the surface of moDCs.

### The comparison of MM patient-moDCs and HD-moDCs

**FIGURE 3:**
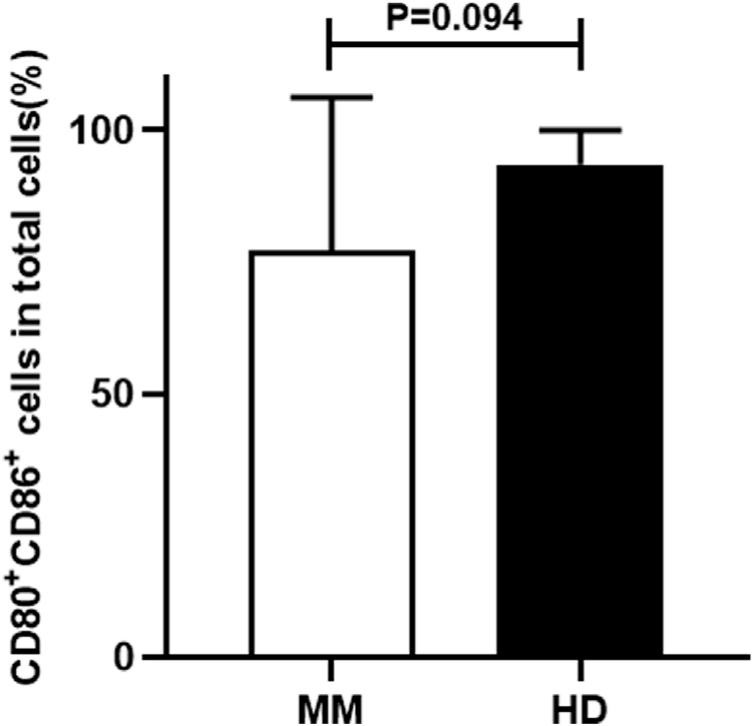
The comparison of the proportion of CD80^+^ CD86^+^ cells in total cells between the MM patient group and the HD group.

We firstly compared the proportion of CD80^+^ CD86^+^ cells in total cells between the MM patient group and the HD group. It was found that the proportion of CD80^+^ CD86^+^ cells in total cells in the HD group was higher than that in the MM patient group, but the difference was not statistically significant (93.49% ± 6.44% vs. 77.04% ± 29.17%, *p* = 0.094) (Figure 3).

### The effect of pomalidomide on HD-moDCs

**FIGURE 4:**
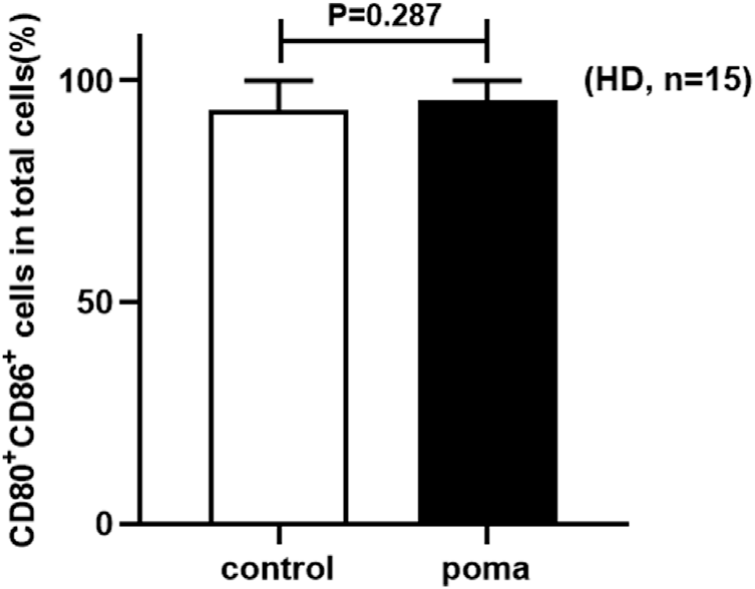
The comparison of the proportion of CD80+ CD86+ cells in total cells between the pomalidomide group and the control group in HDs.

**FIGURE 5:**
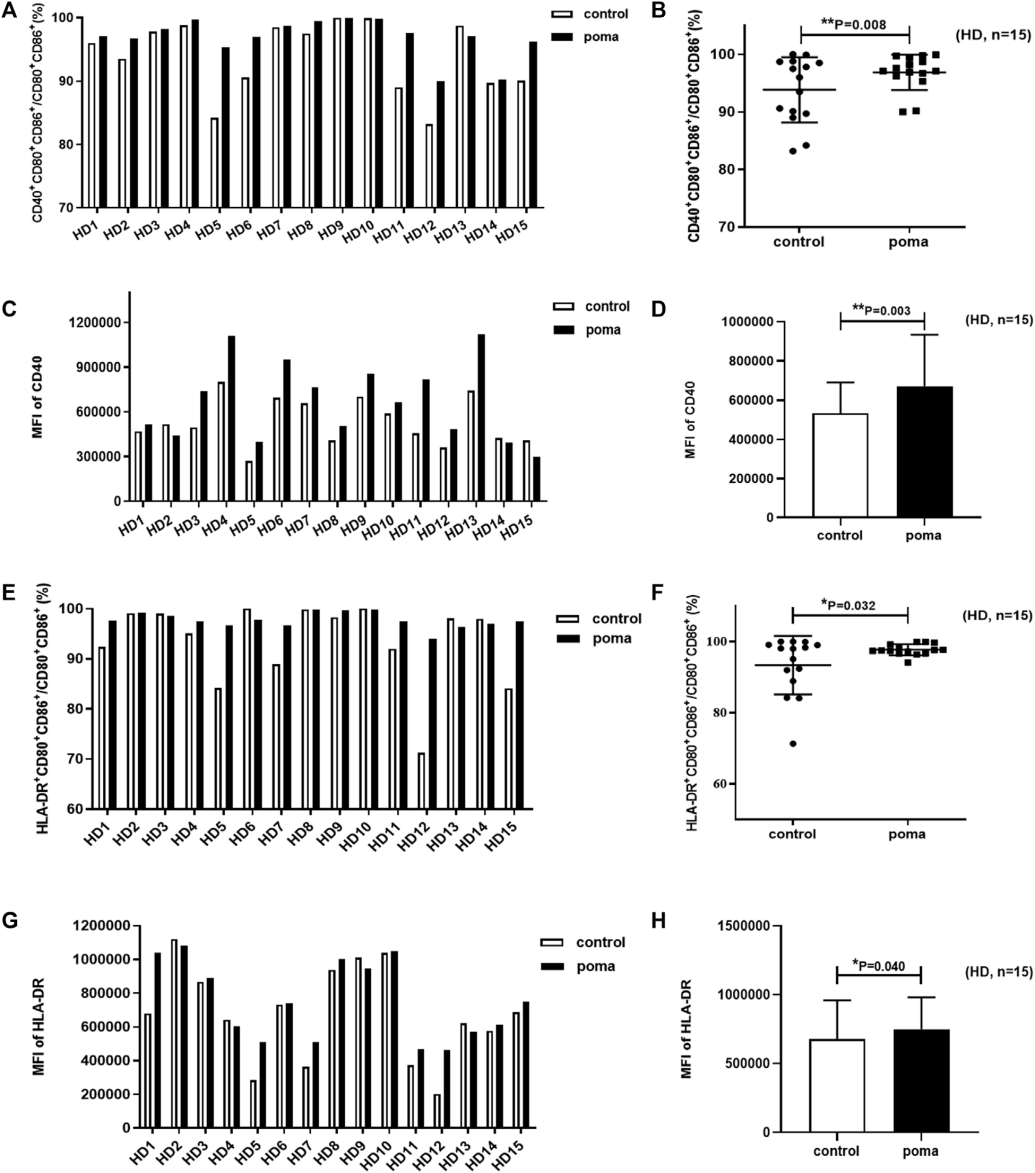
The effect of pomalidomide on the expression of maturation-related surface markers of HD-moDCs (*n* = 15). The proportion of CD40^+^ moDCs in total moDCs was shown in **(A,B)** while the MFI of CD40 expression on moDCs was shown in **(C,D)**. The proportion of HLA-DR^+^ moDCs in total moDCs was shown in **(E,F)** while the MFI of CD40 expression on moDCs was shown in **(G,H)**.

In this study, the differences of HD-moDCs (*n* = 15) between the pomalidomide group and the control group were analyzed. It was found that there was no significant difference in the proportion of CD80^+^ CD86^+^ cells in total cells between the pomalidomide group and the control group (95.42% ± 4.50% vs. 93.49% ± 6.44%, *p* = 0.287) (Figure 4). However, the proportion of CD40^+^ moDCs in total moDCs in the pomalidomide group was significantly higher than that in the control group (96.85% ± 3.08% vs. 93.83% ± 5.67%, *p* = 0.008), and the MFI of CD40 expressed on moDCs in pomalidomide group was also significantly higher than that in the control group (6.70 × 10^5^ ± 2.63 × 10^5^ vs. 5.33 × 10^5^ ± 1.56 × 10^5^, *p* = 0.003) (Figure 5). The proportion of HLA-DR^+^ moDCs in total moDCs in the pomalidomide group was significantly higher than that in the control group (97.73% ± 1.56% vs. 93.36% ± 8.22%, *p* = 0.032), and the MFI of HLA-DR expressed on moDCs in pomalidomide group was also significantly higher than that in the control group (7.49 × 10^5^ ± 2.32 × 10^5^ vs. 6.76 × 10^5^ ± 2.84 × 10^5^, *p* = 0.040) (Figure 5). In conclusion, pomalidomide significantly increases the expression of CD40 (costimulatory molecule) and HLA-DR (MHC-Ⅱ molecular) on HD-moDCs. CD40 and HLA-DR are DC maturation-related surface markers. The results indicate that pomalidomide significantly enhances the maturation of moDCs derived from HDs.

### The effect of pomalidomide on MM patient-moDCs

**FIGURE 6:**
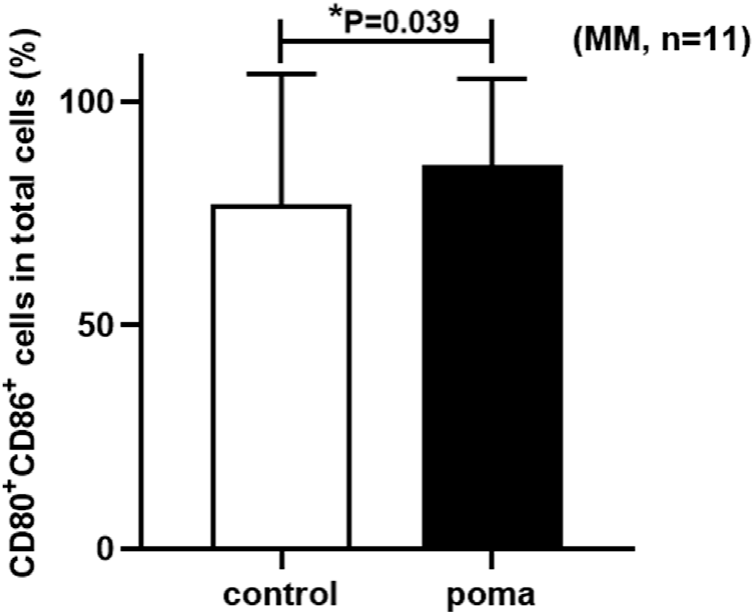
The comparison of the proportion of CD80^+^ CD86^+^ cells in total cells between the pomalidomide group and the control group in MM patients.

**FIGURE 7:**
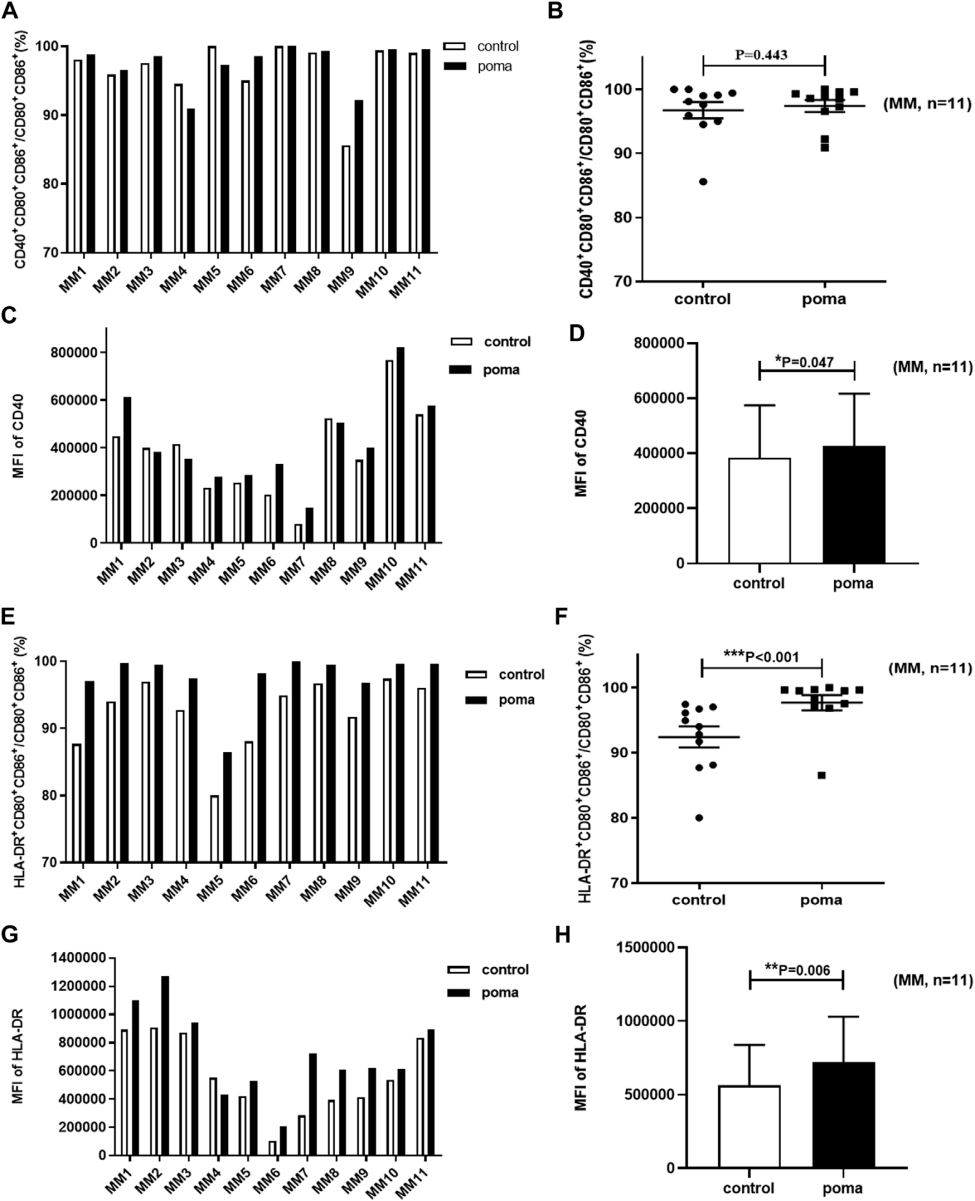
The effect of pomalidomide on the expression of maturation-related surface markers of MM patient-moDCs (*n* = 11). The proportion of CD40^+^ moDCs in total moDCs was shown in **(A,B)** while the MFI of CD40 expression on moDCs was shown in **(C,D)**. The proportion of HLA-DR^+^ moDCs in total moDCs was shown in **(E,F)** while the MFI of HLA-DR expression on moDCs was shown in **(G,H)**.

In this study, the differences of MM patient-moDCs (*n* = 11) between the pomalidomide group and the control group were analyzed. It was found that the proportion of CD80^+^ CD86^+^ cells in total cells in the pomalidomide group was significantly higher than that in the control group (85.68% ± 29.17% vs. 77.04% ± 19.42%, *p* = 0.039) (Figure 6). There was no significant difference in the proportion of CD40^+^ moDCs in total moDCs between the pomalidomide group and the control group (97.4% ± 3.08% vs. 96.75% ± 4.18%, *p* = 0.443), but the MFI of CD40 expressed on moDCs was significantly higher in pomalidomide group than that in the control group (4.27 × 10^5^ ± 1.90 × 10^5^ vs. 3.83 × 10^5^ ± 1.91 × 10^5^, *p* = 0.047) (Figure 7). The proportion of HLA-DR^+^ moDCs in total moDCs in the pomalidomide group was significantly higher than that in the control group (97.65% ± 3.87% vs. 92.4% ± 5.31%, *p* < 0.001), and the MFI of HLA-DR expressed on moDCs in pomalidomide group was also significantly higher than that in the control group (7.23 × 10^5^ ± 3.06 × 10^5^ vs. 5.64 × 10^5^ ± 2.75 × 10^5^, *p* = 0.006) (Figure 7). Overall, pomalidomide significantly increases the expression of CD40 and HLA-DR on MM patient-moDCs. CD40 and HLA-DR are DC maturation-related surface markers. The results suggest pomalidomide significantly enhances the maturation of moDCs derived from MM patients.

### Effect of pomalidomide on cytokines produced by HD-moDCs and MM patient-moDCs

**FIGURE 8:**
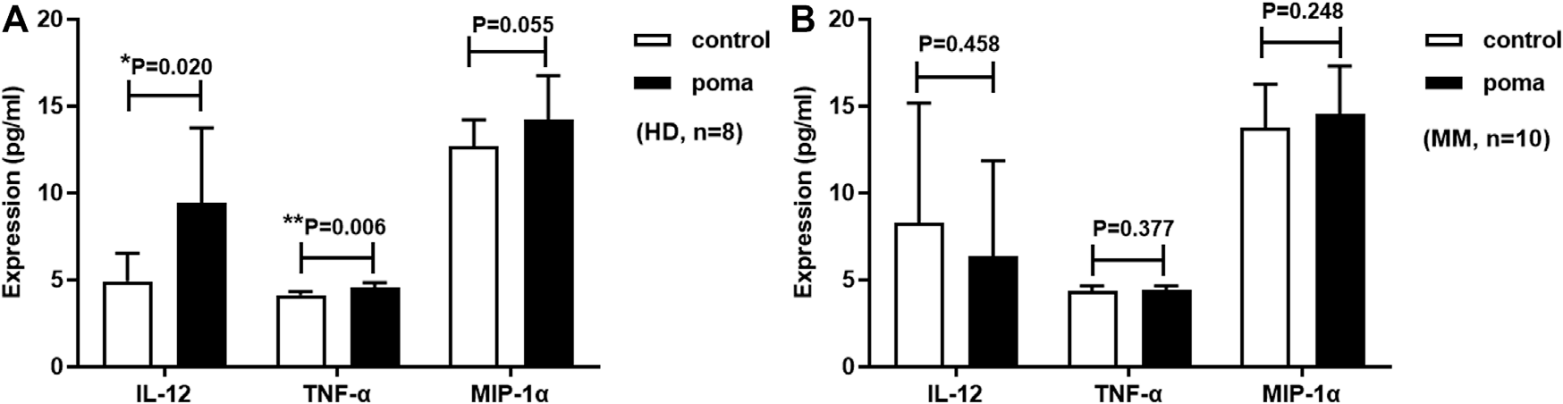
Pomalidomide affects moDC cytokine expression. **(A)** shows that the expression of IL-12 and TNF-α produced by pomalidomide treated HD-moDCs was significantly higher than untreated HD-moDCs (*n* = 8). **(B)** shows that the expression of IL-12, TNF-α, and MIP-1α from MM patient-moDCs showed no significant difference between the pomalidomide group and the control group (*n* = 10).

MoDCs were treated with 10 µM pomalidomide or without pomalidomide, and supernatant from the incubation system was collected on the eighth day. The expression of IL-12, TNF-α, and MIP-1α was analyzed by ELISA. Pomalidomide treated HD-moDCs (*n* = 8) produced 192% IL-12 (9.42 ± 4.31 vs. 4.90 ± 1.61 pg/ml, *p* = 0.020), 110% TNF-α (4.54 ± 0.28 vs. 4.11 ± 0.20 pg/ml, *p* = 0.006) and 112% MIP-1α (14.21 ± 2.54 vs. 12.68 ± 1.53 pg/ml, *p* = 0.055) of untreated moDCs. However, when analyzing MM patient-moDCs (*n* = 10), the expression of IL-12 (6.34 ± 5.51 vs. 8.27 ± 6.88 pg/ml, *p* = 0.458), TNF-α (4.42 ± 0.21 vs. 4.32 ± 0.32 pg/ml, *p* = 0.377) and MIP-1α (14.53 ± 2.76 vs. 13.76 ± 2.48 pg/ml, *p* = 0.248) from moDCs showed no significant difference between pomalidomide group and the control group (Figure 8).

## Discussion

It is well known that not only the cancer cells but also the cancer microenvironment plays an essential role in the oncogenesis, progression, and relapse of cancer diseases (Kawano et al., 2017). The immunosuppressive mechanisms during cancer progression mainly include 1) Expansion of regulatory immune cells such as regulatory T cells, 2) Inhibition of immune effector cells such as effector T cells and NK cells, and 3) Dysfunction of APCs (Pinzon-Charry et al., 2005; Andersen, 2014; Vinay et al., 2015). It is well established that MM patients show significant immune deficiency. Importantly, the DCs in MM patients are dysfunctional (Shinde et al., 2018; Li and Wang, 2019; Tamura et al., 2019). Therefore, it is urgent to search for strategies which can improve the function of DCs in MM patients. In this study, we demonstrated that pomalidomide significantly increases the expression of DC maturation-related surface markers CD40 and HLA-DR on MM-patient moDCs. As pomalidomide significantly enhances the maturation of MM patient-moDCs, pomalidomide is an effective and potential strategy to reverse the DC dysfunction of MM patients.

In our study, we firstly compared the HD-moDCs and MM patient-moDCs. The proportion of CD80^+^ CD86^+^ cells in total harvested cells in the MM group was lower than that in the HD group, indicating that fewer moDCs can be generated from MM patients than from HDs *in vitro*. Notably, when analyzing moDCs from MM patients, we further found that pomalidomide significantly enhanced the proportion of moDCs in total harvested cells. This result suggested that pomalidomide can increase the number of MM patient-moDCs cultured *in vitro*, which lays the foundation for applying DC-based immunotherapy strategies to MM patients. In addition, the CD40 expression on HD-moDCs was significantly higher than that on MM patient-moDCs, but the expression of HLA-DR as well as the secretion of IL-12, TNF-α and MIP-1α don’t show differences between the MM patient group and the HD group (data is not shown). The MM patient donors enrolled in this study all achieved a favorable remission of PR or CR. It is possible that the immune status of these patients has improved with the remission. Thus, further investigations on moDCs from MM patients with different disease stages, such as newly diagnosed MM patients or relapsed MM patients, should be conducted for a comprehensive understanding of the differences between HD-moDCs and MM patient-moDCs.

We then analyzed whether common surface markers of DC maturation were affected by pomalidomide. Pomalidomide induced the significant increases in CD40 and HLA-DR expression on the HD-moDCs and MM patient-moDCs, suggesting pomalidomide can significantly enhance the maturation of moDCs derived from both HDs and MM patients.

Next, we also examined the effect of pomalidomide on the cytokine secretion from DCs to analyze the effect of pomalidomide on the activity/maturation of moDCs. Cytokines secreted by DCs such as interleukin-10 (IL-10), IL-12, interleukin-6 (IL-6), IFN-γ, TNF-α, monocyte chemotactic protein-1 (MCP-1), and MIP-1α can reflect the activity/maturation of DCs (Dudek et al., 2013; Henry et al., 2013; Choi et al., 2018; Gierlich et al., 2020). Among them, the cytokines IL-12, TNF-α, and MIP-1α are most commonly used in many studies, which were detected in this study. HD-moDCs with pomalidomide administration secrete significantly enhanced levels of IL-12 and TNF-α, which indicates that pomalidomide is able to enhance the maturation/activity of HD-moDCs. For those healthy people with high risk of cancer diseases, our results suggest that pomalidomide is potential to enhance the DC maturation/activity of these people, so as to prevent the occurrence of disease or the pre-clinical abnormalities from progression into disease. Moreover, MM patient-derived moDCs with pomalidomide administration secrete enhanced levels of TNF-α and MIP-1α, however, the difference is not statistically significant. This may result from the limited sample size of MM patients. In the future, further research should be conducted with an enlarged sample size of MM patients to demonstrate the effect of pomalidomide on the cytokines secreted by MM patient-moDCs.

In conclusion, our study indicates that pomalidomide enhances the maturation of HD-moDCs and MM patient-moDCs. This mechanism possibly contributes to the total therapeutic efficacy of pomalidomide in MM patients. In addition, pomalidomide shows the potential as a DC adjuvant for application in DC-based therapeutic strategies, such as DC vaccine and DC cell therapy.

## Data Availability

The original contributions presented in the study are included in the article/supplementary material, further inquiries can be directed to the corresponding author.
